# LUM as a novel prognostic marker and its correlation with immune infiltration in gastric cancer: a study based on immunohistochemical analysis and bioinformatics

**DOI:** 10.1186/s12876-023-03075-w

**Published:** 2023-12-21

**Authors:** Wu Xu, Shasha Chen, Qiuju Jiang, Jinlan He, Feifei Zhang, Zhuying Wang, Caishun Ruan, Bin Shi

**Affiliations:** 1 Department of Medical Oncology, Longyan People’s Hospital, No.31 Denggao West Road, Longyan, Fujian 364000 People’s Republic of China; 2 Department of Pathology, Longyan Second Hospital, No.8 Shuangyang West Road, Longyan, Fujian 364000 People’s Republic of China

**Keywords:** Gastric cancer, LUM, Prognosis, miRNA, lncRNA, Tumour infiltration

## Abstract

**Background:**

Gastric cancer (GC) is considered the sixth highly prevailing malignant neoplasm and is ranked third in terms of cancer mortality rates. To enable an early and efficient diagnosis of GC, it is important to detect the fundamental processes involved in the oncogenesis and progression of gastric malignancy. The understanding of molecular signaling pathways can facilitate the development of more effective therapeutic strategies for GC patients.

**Methods:**

The screening of genes that exhibited differential expression in early and advanced GC was performed utilizing the Gene Expression Omnibus databases (GSE3438). Based on this, the protein and protein interaction network was constructed to screen for hub genes. The resulting list of hub genes was evaluated with bioinformatic analysis and selected genes were validated the protein expression by immunohistochemistry (IHC). Finally, a competing endogenous RNA network of GC was constructed.

**Results:**

The three genes (ITGB1, LUM, and COL5A2) overexpressed in both early and advanced GC were identified for the first time. Their upregulation has been linked with worse overall survival (OS) time in patients with GC. Only LUM was identified as an independent risk factor for OS among GC patients by means of additional analysis. IHC results demonstrated that the expression of LUM protein was increased in GC tissue, and was positively associated with the pathological T stage. LUM expression can effectively differentiate tumorous tissue from normal tissue (area under the curve = 0.743). The area under 1-, 3-, and 5-year survival relative operating characteristics were greater than 0.6. Biological function enrichment analyses suggested that the genes related to LUM expression were involved in extracellular matrix development-related pathways and enriched in several cancer-related pathways. LUM affects the infiltration degree of cells linked to the immune system in the tumor microenvironment. In GC progression, the AC117386.2/hsa-miR-378c/LUM regulatory axis was also identified.

**Conclusion:**

Collectively, a thorough bioinformatics analysis was carried out and an AC117386.2/hsa-miR-378c/LUM regulatory axis in the stomach adenocarcinoma dataset was detected. These findings should serve as a guide for future experimental investigations and warrant confirmation from larger studies.

**Supplementary Information:**

The online version contains supplementary material available at 10.1186/s12876-023-03075-w.

## Introduction

Gastric cancer (GC) is the sixth highest prevailing cancer in humans with the third highest death rate [1]. Although there have been advancements in prevention ad diagnosis as well as a prompt and effective treatment, the number of new GC cases and deaths continue to rise. Global estimates for the year 2020 show that GC will have an incidence number of 1,089,103 and a mortality number of 768,793 [1]. Most GC patients had advanced or metastatic disease when first diagnosed due to nonspecific symptoms and a lack of suitable biomarkers [2]. Clinical evidence also suggests that patients with early-stage GC have a relatively favorable outcome, with a 5-year disease-specific survival rate of 90%. In contrast, individuals with advanced cancer (stage IV) typically have a dismal prognosis, with a 5-year disease-specific survival rate of 5–15% [3–5]. Conventional prognostic factors, such as pathological grade and tumor-node-metastasis (TNM) stage, are inadequate to accurately predict patients’ prognosis [6, 7]. Therefore, finding prospective GC targets and prognostic biomarkers is one of the most common research areas.

Cancer is a heterogeneous and complex disease characterized by genetic alterations that accumulate during tumor initiation, development, and progression. Recently, high-throughput sequencing technology and genomic microarrays based on large cohorts have emerged as potentially powerful tools for elucidating the mechanisms of disease biomarkers and related pathways [8]. For example, Zhang et al. investigated the genome-wide micro RNA (miRNA) expression profile of individuals with GC and identified relevant molecules has-miR-16-5p and has-miR-19b-3p, thereby offering novel directions for diagnosing and effectively treating GC [9]. Zhu et al. demonstrated that a disintegrin and metalloprotease 12 (ADAM12) may act as a tumor promoter of gastric cancer through the integrated bioinformatics methods and experimental analyses [10]. Furthermore, Wang and colleagues found that tissue inhibitor of metalloproteinase-2 (TIMP2) could act as a novel candidate biomarker in GC patients using bioinformatics analysis, and they revealed the potential molecular mechanism of TIMP2 in GC malignant progression [11]. Although these studies identified gene expression patterns that may aid in the diagnosis and prognosis of GC, these approaches did not account for the different stages or subtypes of GC. Therefore, it was needed to identify novel molecular biomarkers that stratify GC.

**Fig. 1:**
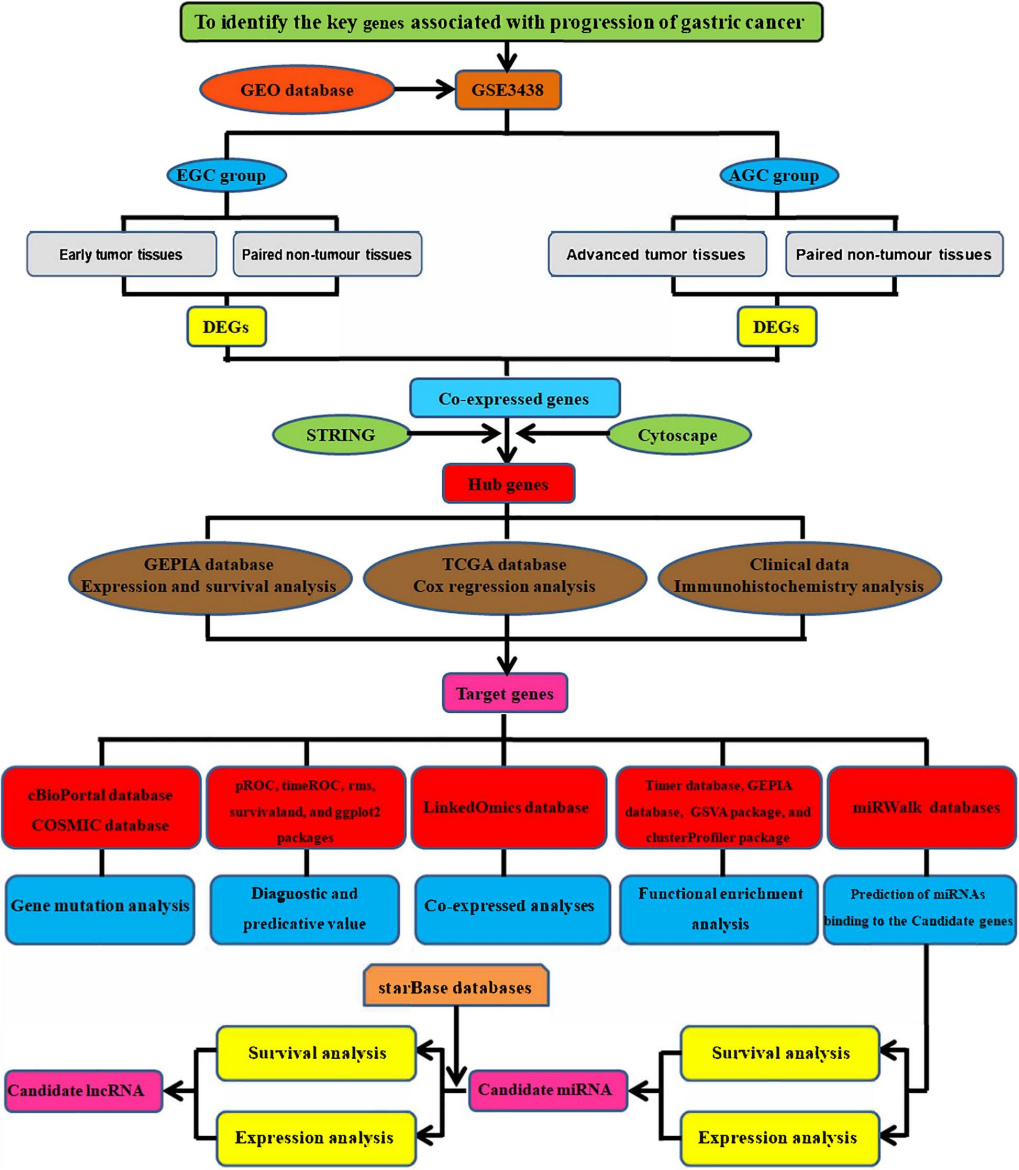
Flow chart of this research

Accordingly, in the present research, a network linked to clarify the potential mechanism of GC progression was analytically developed (Fig. 1). First, the differentially expressed genes (DEGs) linked to gastric carcinogenesis and cancer progression were detected using the Gene Expression Omnibus (GEO) dataset, GSE3438. Afterward, the hub genes were filtered out by analyzing the protein–protein interaction (PPI) network through the software, Cytoscape, and the database, the Search Tool for the Retrieval of Interacting Genes (STRING) with both the tools utilizing DEGs. Simultaneously, the database, Gene Expression Profiling Interactive Analysis (GEPIA) was utilized to validate the DEGs contained in the study, and the genes with differential expression that showed considerable association with overall survival (OS) were retained. The independent prognosis-linked factors for OS were assessed utilizing the Cox regression function. Simultaneously, the protein production of target genes in clinically collected GC tissues was determined by immunohistochemistry (IHC). Subsequently, the diagnostic and predictive value of target genes was evaluated. The target genes were also analyzed for enrichment analysis of the Kyoto Encyclopedia of Genes and Genomes (KEGG) pathway, co-expression, Gene Ontology (GO) function, Gene Set Enrichment Analysis (GSEA), genetic alterations, and tumor immunology. Lastly, to elucidate the potential mechanism of the target gene in GC at the molecular level, a network of messenger RNA (mRNA)-microRNA (miRNA)-long non-coding RNA (lncRNA) interactions was designed. The development of effective therapeutic targets for GC and the growth in the pool of prognosis-linked markers can be a potential benefit of the findings of this research.

## Materials and methods

### Clinical samples

A total of 46 patients diagnosed with GC were included in this study, comprising 9 cases of early-stage cancer and 37 cases of advanced cancer. The patients underwent surgical resection or gastroscopy biopsy at Longyan People's Hospital in Fujian Province, China, between January 2021 and December 2022. The study excluded patients who had undergone preoperative radiotherapy and/or chemotherapy. Table 1 displays the clinical manifestations of the patients. All gastric cancer specimens were histologically classified by two pathologists according to the eighth edition of the American Joint Committee on Cancer (AJCC) TNM staging system. Tumor invasion into the mucosa and/or submucosa was classified as early-stage cancer. In contrast, tumor infiltration into the intrinsic muscular layer, subserosa, serosal layer, and adjacent tissues was defined as advanced cancer, irrespective of lymph node metastasis. In the control group, specimens were collected from the paracancerous tissues of the same patients, located at least 2 cm away from the cancerous tissue. Both clinical and control specimens were fixed in 10% formalin for 24 h at room temperature and placed in an automatic dehydration machine (Tianli Aviation Electrical Co. Ltd., Tianjin, China). Subsequently, fixed tissues were manually embedded in liquid paraffin to make the paraffin blocks and sectioned longitudinally (4 μm sections). Prior to the study, written informed consent was obtained from all patients to use their clinical specimens, and the Institutional Review Board of the Longyan People's Hospital approved the use of all tissues and clinical information with approval number 2020–023.

**Table 1:** Clinical features of the studied patients with gastric cancer

Clinical Characteristic	levels	N (46)	Percentage (%)
Age	≥ 60	31	67.4
	< 60	15	32.6
Gender	Male	21	45.7
	Female	25	54.3
TNM stage	I	15	32.6
	II	22	47.8
	III	5	10.9
	IV	4	8.7
T stage	T1	9	19.6
	T2	16	34.8
	T3	14	30.4
	T4	7	15.2
N stage	N0	21	45.6
	N1	17	37.0
	N2	8	17.4
	N3	0	0
M stage	M0	42	91.3
	M1	4	8.7

### Microarray data

The mRNA expression microarrays of GC samples were accessed at the database, GEO (http://www.ncbi.nlm.nih.gov/geo) using keywords (“gastric cancer” OR “stomach carcinoma” (all fields) AND “*Homo sapiens*” (porgn). Subsequently, the relevant datasets were filtered out by studying the titles and abstracts, and complete information about the relevant datasets was evaluated. The following are the criteria for final dataset selection: (1) the study cohort included samples with normal tissues adjacent to a tumor (or precancerous tissues); (2) the study cohort included at least ten tumor samples and ten non-tumor samples; and (3) the study cohort included both early-stage and advanced-stage GC. Ultimately, a publicly available dataset, GSE3438, was made a part of this research [12]. The GSE3438, created on the Center for Functional Analysis of the Human Genome Human KUGI 14 K platform, contained 49 pairs of tissue samples, including 10 pairs of early-stage GC (EGC) samples and 39 pairs of advanced-stage GC (AGC) samples. Each sample was divided into the paired tumor and normal tissues.

### Differential expression analysis

The DEGs were accessed at GEO using the GEO2R (http://www.ncbi.nlm.nih.gov/geo/geo2r) web application. Initially, two groups of samples were established: EGC and AGC. In GSE3438, principal component analysis (PCA) was employed to assess the repeatability of sample data. The differential expression analysis was repeated separately in early and late GC patients. The criteria for inclusion were |log2FC|> 1 (FC = fold change) and an adjusted *P*‐value < 0.05. Finally, the DEGs were analyzed, and custom Venn diagrams were created to compare the DEGs in two datasets. The DEGs in the “EGC” group and the “AGC” group were classified in comparison with the associated paired adjacent non-tumor tissues as upregulated or downregulated and genes were termed as significant DEGs.

### PPI network construction and hub genes screening

A PPI network concerning the DEGs was generated utilizing STRING 11.5 (Szklarczyk et al*.,* 2011) (https://string-db.org/) with a confidence cut-off of 0.4 [13]. Cytoscape 3.9.1 was employed to analyze and process this network [14]. The Maximal Clique Centrality (MCC) algorithm in the Cytoscape cytoHubba application was utilized to identify hub genes in potential targets.

### Hub gene expression, OS and pathologic stage analysis

The newly developed interactive tool, GEPIA 2 (Tang et al*.,* 2017) website (http://gepia2.cancer-pku.cn/#analysis), was utilized for the profiling of the gene expression and interactive analyses of tumor tissues and non-cancerous tissue samples as per the Cancer Genome Atlas (TCGA) and Genotype-Tissue Expression (GTEx) project [15, 16]. The TCGA and GTEx databases were utilized to assess the relative expression levels of hub genes in this study. In GEPIA, “*” denotes a |Log2FC| cutoff value > 1 and a *P*-value cutoff < 0.05. Moreover, the hub genes in GC were analyzed utilizing the GEPIA tool for their OS values and then those genes with statistical significance were screened out. Finally, in the GEPIA, the candidate genes whose expression was considerably linked to survival were explored in the pathological stage-specific pattern.

### Clinical and bioinformatic information

The expression profiles of 407 GC samples (transcripts per million, TPM), along with the clinical follow-up data, were accessed at the TCGA stomach adenocarcinoma (STAD) dataset (February 17, 2022, https://portal.gdc.cancer.gov/projects/TCGA-STAD) for subsequent candidate gene analyses. These samples included tissues of tumorous nature that numbered 375 and paracancerous tissue that numbered 32, which included 27 pairs of GC tissues and their matched non-tumor tissue samples. As the database was publicly accessible to researchers online, approval from the local ethics committee was not mandatory for this research. Univariate Cox regression was employed to validate candidate genes in the TCGA-STAD cohort. Concurrently, the selection of clinical factors linked to prognosis (*P* < 0.05) was carried out using the univariate Cox regression model. Using multivariate Cox regression, the candidate genes and clinical parameters with a *P*-value < 0.05 were selected where the genes with a *P*-value < 0.05 were designated as prognosis-related factors (target genes) for subsequent analysis.

### IHC detection

IHC was employed to determine the protein expression of target genes in specimens from subjects with gastric cancer and non-tumor controls. IHC staining was performed using an Avidin–Biotin-Enzyme Complex (ABC) kit (catalog number 32020; Thermo Scientific, USA) in accordance with guiding instructions from the manufacturer. Two pathologists who were not privy to clinical data subjected tissue samples to lighting through a microscope (Olympus Corporation), following which they were graded blind to determine their overall stain intensity into one of four categories: negative (0), weak (1), moderate (2), or strong (3). In each section, the percentage of cells that had positive stains was evaluated in five distinct fields at magnification × 400, with values ranging from 0 to 100%. The degree of staining was categorized as 0 (< 10%), 1 (10–30%), 2 (30–50%), and 3 (50–100%). Based on the sum score (staining intensity added to the positive cell score), we categorized the expression into four tiers: negative (-, score 0); weakly positive (± , scores 1–2); positive (+ , scores 3–4); and strongly positive (+ + , scores 5–6). For immunochemical tests, we employed anti-Lumican (LUM) antibodies (catalog no. ab168348, dilution 1:100; Abcam, Shanghai, China).

### Gene mutation analysis

The cBioPortal database (http://cbioportal.org, Cerami et al., 2012) offers a platform for the exploration, visualization, and analysis of diverse cancer genomic data [17]. Using the cBioPortal database, we analyzed the gene mutation status of the target genes in the data of TCGA PanCancer Atlas Studies. The Catalogue of Somatic Mutations (COSMIC) database (http://www.sanger.ac.uk/cosmic/, Forbes et al., 2010) is an additional public resource providing information on somatic mutations in human cancers for specific input genes [18]. The mutation types and frequency of LUM were obtained from the COSMIC.

### Functional enrichment analysis

The database, LinkedOmics (http://www.linkedomics.org/login.php, Vasaikar, et al*.,* 2018) is a platform that can be accessed by the public that currently analyzes the multi-omics data from the TCGA database, containing 32 cancer types [19]. In this study, the genes that showed variation in their expression data related to the target gene were filtered out utilizing the LinkFinder module from the TCGA-STAD cohort. The Pearson correlation coefficient was employed for the purpose of performing the search results that were displayed as a heat map and a volcano map. The (false discovery rate (FDR) < 0.01 was set as the rank criterion. Otherwise, to observe the effect of target gene expression on tumors, the samples of the TCGA-STAD dataset were categorized into high-risk (50%) and low-risk (50%) groups according to gene expression levels, and the enrichment of KEGG and HALLMARK pathways in the high and low expression group was analyzed using GSEA. The gene sets h.all.v7.5.1.symbols.gmt and c2.cp.kegg.v7.5.1.symbols.gmt were chosen as the reference gene set. The nominal *P*-value < 0.05, normalized enrichment score (|NES|> 1), and FDR q-value < 0.25 were considered as significant pathway enrichment.

### Tumor immunology analysis

The tumor immune estimation resource (TIMER) (https://cistrome.shinyapps.io/timer/, Li et al*.,* 2017), which relies on TCGA gene expression profiles, is a validated and reliable database [20]. The infiltration level of the cells linked to the immune system can be assessed, and their potential clinical effect can be observed utilizing the TIMER tool. The Gene module was utilized to examine the link between target gene transcription levels and the abundance of immune cell infiltrates in STAD while the Survival module was employed to generate the Kaplan–Meier (K-M) plots for the target gene and immune infiltrates to determine differences in survival. The comparison of the tumor infiltration levels in tumors with varying somatic copy number alterations (SCNA) for the target gene in STAD was carried out. Furthermore, TIMER was employed to thoroughly examine the link between the target gene and specific immune infiltrating cell subset markers to understand the target gene’s potential role in tumor immunity. The TIMER analysis gene correlations were validated using the GEPIA database.

### Competing endogenous RNA (ceRNA) network construction

A regulatory network of the ceRNA was generated to elucidate the potential function of the target gene in STAD. Initially, the miRWalk (http://129.206.7.150/, Sticht, et al*.,* 2018) database was employed to predict gene-related upstream miRNA [21]. miRWalk integrates various variables of multiple miRNA-specific target prediction programs to provide information on the predicted and validated miRNA binding sites of human miRNAs. The screening criteria were set as bindingp = 1, energy < -20, accessibility < 0.01 and au > 0.5 [22]. Based on the above analysis, these predicted miRNAs were confirmed as candidate miRNAs for candidate genes. StarBase (http://starbase.sysu.edu.cn/, version 3, Li et al*.,* 2014) was utilized to predict lncRNA targets interacting with miRNAs based on the miRNAs identified [23, 24]. The StarBase database is open-source for studying non-coding RNAs such as microRNA/ lncRNA/circRNA. The TCGA-STAD dataset was utilized to analyze miRNA and lncRNA targets’ expression and prognostic values (OS).

### Statistical analysis

R 3.6.3 software was employed for data preparation and analysis. The one-way analysis of variance (one-way analysis of variance), as well as the Wilcoxon rank-sum test, or Student’s t-test were utilized for comparisons between groups, as applicable. Survival curves (K-M) were plotted and logarithmic tests were done to determine the significance of differences between the two survival curves. The independent prognostic parameters that were of significance were identified using single and multivariate Cox hazard regression analyses utilizing a stepwise approach. The rank correlation analysis was used for correlation analysis (Spearman). The diagnostic relative operating characteristics (ROC) curve, time-dependent survival ROC curve, and nomogram model analysis were plotted using R packages, including the pROC, timeROC, rms, survival, and ggplot2 packages. The “clusterProfiler” package was utilized to perform functional enrichment analysis of the potential target, including GO, KEGG (www.kegg.jp/kegg/kegg1.html) [25], and GSEA. Immune infiltration analysis of LUM was performed by single sample Genome Enrichment Analysis (ssGSEA) using the gene set variation analysis (GSVA) R software package. Significant criterion was considered to be *P*-value < 0.05. In figures the statistical significance was considered to be * *P* < 0.05, ** *P* < 0.01, and *** *P* < 0.001.

## Results

### Candidate genes screening for GC stage progression

**Fig. 2:**
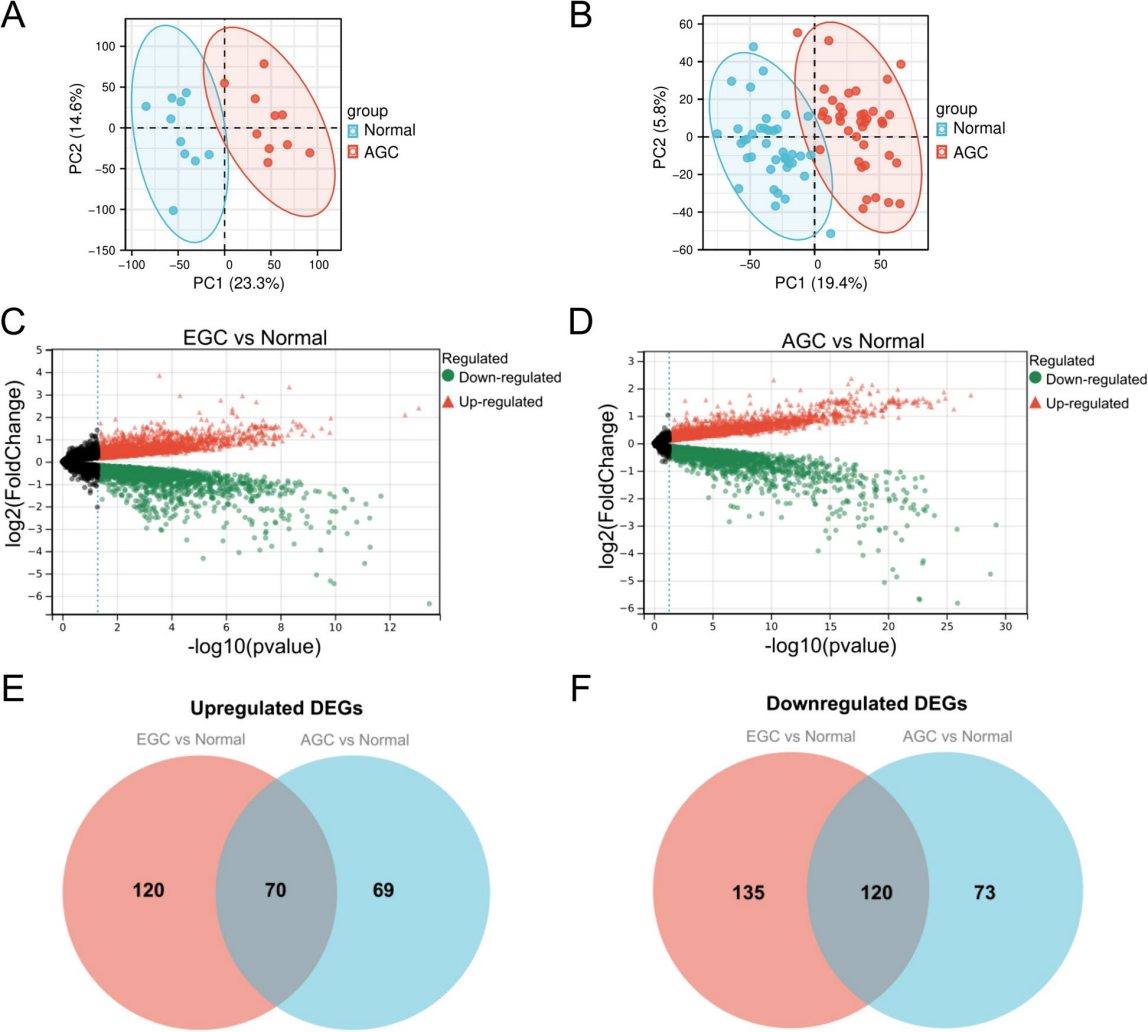
The differentially expressed genes (DEGs) between the paired tumor and normal gastric tissues were detected. **A** Principal component analysis for the group of early-stage GC (EGC). **B** Principal component analysis for the group of advanced-stage GC (AGC). **C** Volcano plot of DEGs between EGC and paired para-tumor tissues. **D** Volcano plot of DEGs between AGC and paired para-tumor tissues. Note: the black data points represent genes that are not considerably changed, and the red data points and green data points signify the upregulated and downregulated genes in early GC (in comparison with paired para-tumor tissues) and advanced GC (in comparison with paired para-tumor tissues), respectively. |log2FC|> 1 and adj *P* < 0.05 were selected as the cut-off criteria. FC = fold change. **E** The intersection of upregulated DEGs of “EGC vs. Normal” and “AGC vs. Normal”. **F** The intersection of downregulated DEGs of “EGC vs. Normal” and “AGC vs. Normal.” “EGC vs. Normal” represents the differential expression analysis between EGC tissues and paired non-tumor tissues. “AGC vs. Normal” represents the differential expression analysis between AGC tissues and paired non-cancerous tissues

PCA was employed to examine the repeatability of intra-group data. The analysis depicted that the intra-group data repeatability in GSE3438 is satisfactory between EGC tissues and paired adjacent non-tumor tissues, as well as in the AGC tissues and paired adjacent non-tumor tissues (Fig. 2A, B). The GSE3438 data series was then analyzed using the GEO2R algorithm to screen for key genes involved in GC progression. Many DEGs were discovered between the “EGC” and “Normal” groups or between the “AGC” and “Normal” groups (Fig. 2C, D). As per the threshold criteria utilized, a set of 445 DEGs in total were detected in EGC samples, compared to paired non-tumor tissues, in which upregulation and downregulation of 190 and 255 DEGs were detected respectively (Supplementary file 1). Similarly, a set of 332 DEGs were detected in AGC samples, compared to paired non-tumor tissues, in which upregulation and downregulation of 139 and 193 DEGs were detected, respectively (Supplementary file 1). This research sought to identify genes closely related to GC progression. Following that, the frequently appearing upregulated and downregulated DEGs in the two comparison sets were obtained. The number of identified DEGs that showed upregulation and downregulation numbered 70 and 120, respectively (Fig. 2E, F and Supplementary file 1). The 190 total DEGs were designated as candidate genes and additional analysis was carried out.

### PPI network analysis and hub gene selection

**Fig. 3:**
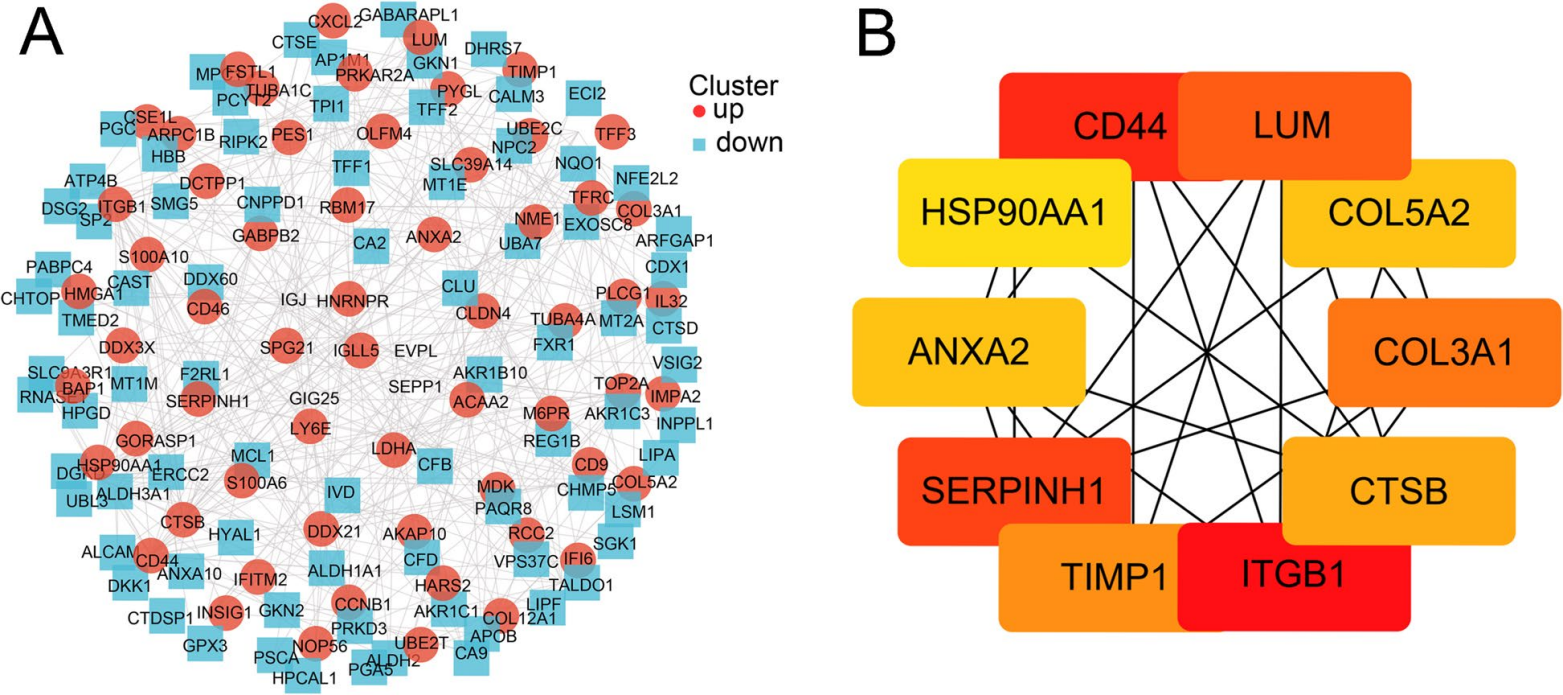
The protein–protein interaction (PPI) network and the most significant modules of differentially expressed genes. **A** A PPI network was generated using Cytoscape (combined score ≥ 0.4). **B** Hub genes were screened from the PPI network using the Maximal Clique Centrality algorithm

STRING constructed a PPI network of DEGs to explore their most significant clusters. Following this period, the analysis file is re-introduced to Cytoscape 3.9.1 software for visual representation (Fig. 3A). Using the MCC algorithm of the cytoHubba plug-in the top ten hub genes were identified: ITGB1, CD44, SERPINH1, LUM, COL3A1, TIMP1, CTSB, COL5A2, ANXA2, and HSP90AA1 (Fig. 3B and Table 2). The hub genes were depicted in darker colors in the figure: ITGB1, CD44, SERPINH1, and LUM. Besides, the levels of expression of all genes in the module were found to be upregulated.

**Table 2:** Top 10 in Network Ranked by MCC Method

Gene symbol	Gene description	Type	Score
ITGB1	Integrin subunit β1	up	164
CD44	Cluster of differentiation 44	up	122
SERPINH1	Serpin peptidase inhibitor clade H, member 1	up	121
LUM	Lumican	up	100
COL3A1	Collagen type III alpha 1 chain	up	96
TIMP1	Tissue inhibitor of metalloproteinase 1	up	82
CTSB	Cathepsin B	up	80
ANXA2	Annexin A2	up	72
COL5A2	Collagen type V alpha 2 chain	up	72
HSP90AA1	Heat shock protein 90-alpha	up	59

*MCC* Maximal clique centrality

### Correlation between hub gene expression, OS and pathologic stage

**Fig. 4:**
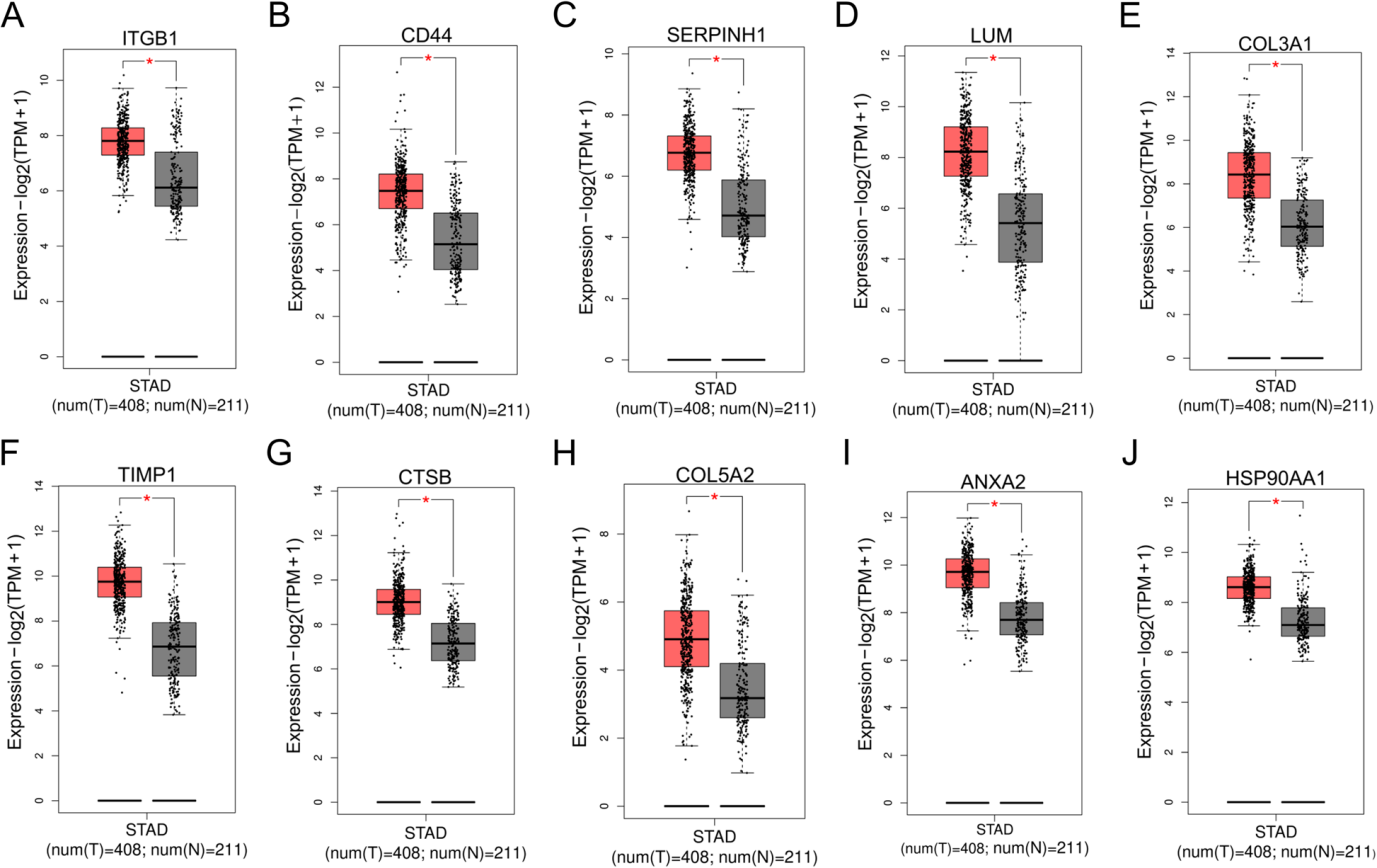
Expression levels of ten hub genes in the tumor (408 samples) in comparison with normal tissue (211 samples). **A** ITGB1, **B** CD44, **C** SERPINH1, **D** LUM, **E** COL3A1, **F** TIMP1, **G** CTSB, **H** COL5A2, **I** ANXA2, and **J** HSP90AA1. “*” represents “*P*-value < 0.05.” Y axis indicates the relative expression value, log2(TPM + 1). TPM = transcripts per million

**Fig. 5:**
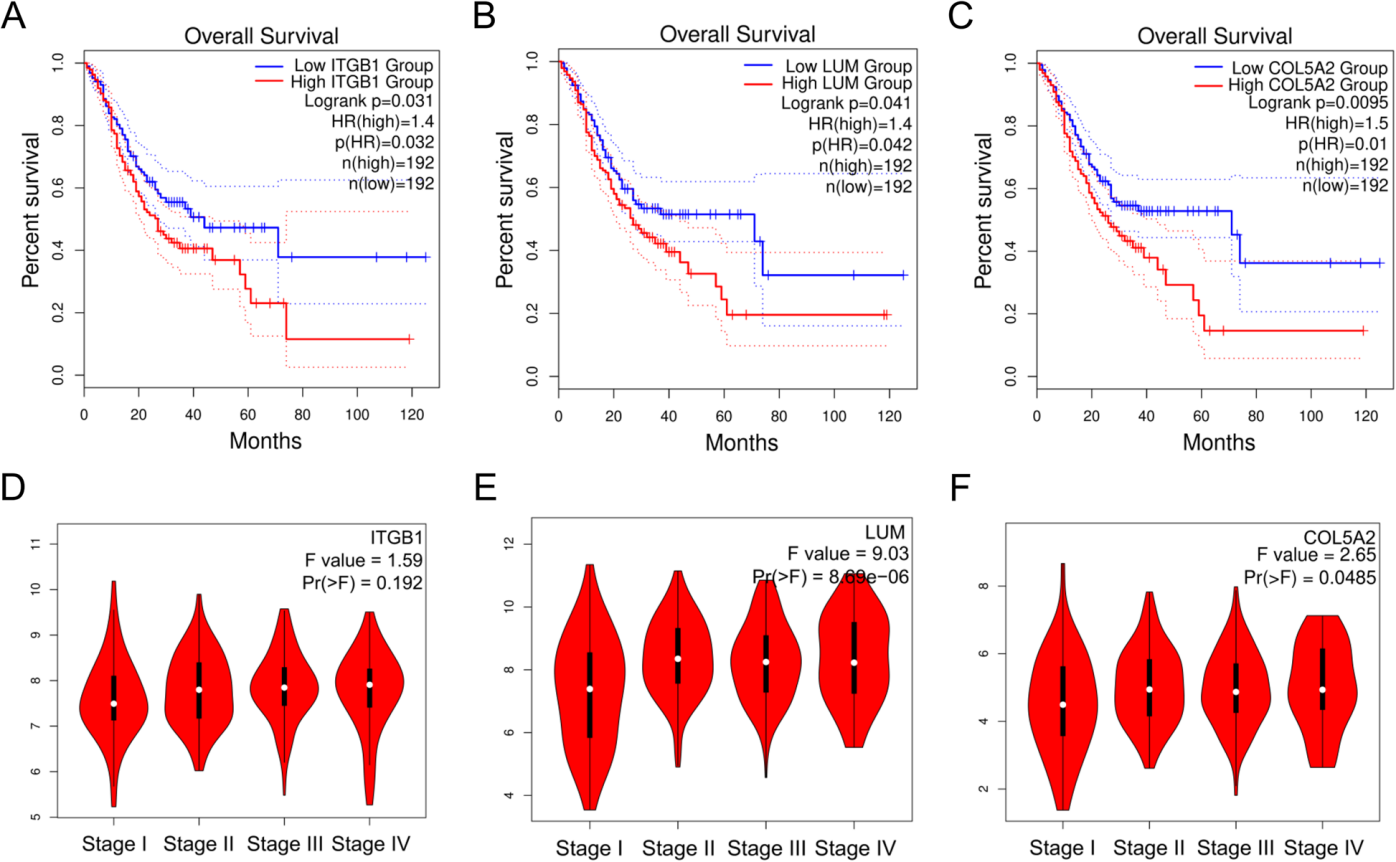
The influence of hub gene expression on the overall survival (OS) and pathologic stage. Survival curves of OS between low and high expression of ITGB1 (**A**), LUM (**B**), and COL5A2 (**C**) in patients with stomach adenocarcinoma (STAD). Relative expression levels of ITGB1 (**D**), LUM (**E**), and COL5A2 (**F**) in STAD of different pathological stages

Ten hub genes were compared concerning their expression in STAD (408 samples) and normal (211 samples) tissues in the GEPIA database to better understand their roles in STAD. The analysis suggested that all ten hub genes were expressed at a considerably elevated level in STAD tissues in comparison with non-cancerous tissues (Fig. 4). Subsequently, using the GEPIA database, the K-M curve and log-rank test analysis were executed to examine the OS for the top ten hub genes. The critical point was considered as the median expression level, and the STAD specimens were categorized accordingly into high and low groups as per their expression levels. The OS analysis depicted that patients with elevated expression levels of ITGB1 (Fig. 5A), LUM (Fig. 5B), and COL5A2 (Fig. 5C) genes had a shorter survival time. In contrast, the remaining seven genes were not significantly associated with OS. ITGB1, LUM, and COL5A2 genes may be associated with GC progression and have clinical prognostic significance for GC. Subsequently, it was discovered that the ITGB1 gene expression (Fig. 5D) was not linked to the pathologic stage of STAD. In contrast, the expression of the LUM (Fig. 5E) and COL5A2 (Fig. 5F) genes was significantly (*P* < 0.05) positively related to the pathologic stage in STAD patients.

### TCGA database patient characteristics

Our data set was compiled from TCGA public available database, including 375 STAD patients with clinical data and gene expression data. The clinical characteristics, including gender, age, T stage, N stage, M stage, pathological stage, primary therapy outcome, residual tumor, histologic grade, *Helicobacter pylori* infection, and gene expression data were collected (Table 3).

**Table 3:** Clinical characteristics of the stomach adenocarcinoma patients in TCGA

Clinical Characteristic	levels	N (375)	Percentage (%)
Gender	Female	134	35.7
	Male	241	64.3
Age	< = 65	164	44.2
	> 65	207	55.8
T stage	T1	19	5.2
	T2	80	21.8
	T3	168	45.8
	T4	100	27.2
N stage	N0	111	31.1
	N1	97	27.2
	N2	75	21
	N3	74	20.7
M stage	M0	330	93
	M1	25	7
Pathologic stage	Stage I	53	15.1
	Stage II	111	31.5
	Stage III	150	42.6
	Stage IV	38	10.8
Primary therapy outcome	PD	65	20.5
	SD	17	5.4
	PR	4	1.3
	CR	231	72.9
Residual tumor	R0	298	90.6
	R1	15	4.6
	R2	16	4.9
Histologic grade	G1	10	2.7
	G2	137	37.4
	G3	219	59.8
H pylori infection	No	145	89
	Yes	18	11
Age, median (IQR)		67 (58, 73)	67 (58, 73)

*TCGA* The Cancer Genome Atlas, *T* Tumor, *N* Node, *M* Metastasis, *PD* Progressive disease, *SD* Stable disease, *PR* Partial response, *CR* Complete response, *IQR* Inter quartile range

### Univariate and multivariate Cox regression analyses

The univariate and multivariate Cox regression analyses of candidate genes and clinical characteristics in the cohort TCGA-STAD were carried out. After the univariate Cox regression analysis, it was found that several factors such as age, pathologic stage, T stage, N stage, M stage, primary therapy outcome, residual tumor, ITGB1, LUM, and COL5A2 had a significant prognostic correlation with OS (Table 3). Subsequently, age (hazard ratio (HR) = 1.571, 95% confidence interval (CI): 1.029–2.398, *P* = 0.036), N stage (HR = 2.023, 95% CI: 1.022–4.004, *P* = 0.043), primary therapy outcome (HR = 4.130, 95% CI: 2.624–6.500, *P* < 0.001), and high LUM expression (HR = 1.830, 95% CI: 1.045–3.203, *P* = 0.034) were considered to be independent prognosis-associated parameters for OS through multivariate Cox regression analysis (Table 4).

**Table 4:** Risk factors for overall survival according to Cox proportional hazards regression model

Characteristics	Total(N)	Univariate analysis		Multivariate analysis	
		Hazard ratio (95% CI)	*P* value	Hazard ratio (95% CI)	*P* value
**Age**	367				
< = 65	163	Reference			
> 65	204	1.620 (1.154–2.276)	**0.005**	1.571 (1.029–2.398)	**0.036**
**Pathologic stage**	347				
Stage I	50	Reference			
Stage II&Stage III&Stage IV	297	2.247 (1.210–4.175)	**0.010**	1.004 (0.357–2.826)	0.994
**T stage**	362				
T1	18	Reference			
T2&T3&T4	344	8.829 (1.234–63.151)	**0.030**	1,533,097.7526 (0.000-Inf)	0.995
**N stage**	352				
N0	107	Reference			
N1&N2&N3	245	1.925 (1.264–2.931)	**0.002**	2.023 (1.022—4.004)	**0.043**
**M stage**	352				
M0	327	Reference			
M1	25	2.254 (1.295–3.924)	**0.004**	1.300 (0.575–2.943)	0.528
**Histologic grade**	361				
G1	10	Reference			
G2&G3	351	1.957 (0.484–7.910)	0.346		
**Primary therapy outcome**	313				
CR	229	Reference			
PD&SD&PR	84	4.228 (2.905–6.152)	** < 0.001**	4.130 (2.624–6.500)	** < 0.001**
***Helicobacter pylori* infection**	162				
No	144	Reference			
Yes	18	0.650 (0.279–1.513)	0.317		
**Residual tumor**	325				
R0	294	Reference			
R1&R2	31	3.445 (2.160–5.494)	** < 0.001**	1.169 (0.613–2.228)	0.636
**ITGB1**	370				
Low	184	Reference			
High	186	1.404 (1.008–1.956)	**0.045**	1.103 (0.695–1.751)	0.676
**LUM**	370				
Low	185	Reference			
High	185	1.433 (1.028–1.997)	**0.034**	1.830 (1.045–3.203)	**0.034**
**COL5A2**	370				
Low	186	Reference			
High	184	1.496 (1.073–2.084)	**0.017**	0.974 (0.571–1.662)	0.923

*T* Tumor, *N* Node, *M* Metastasis, *PD* Progressive disease, *SD* Stable disease, *PR* Partial response, *CR* Complete response

### The protein expression level of LUM

**Fig. 6:**
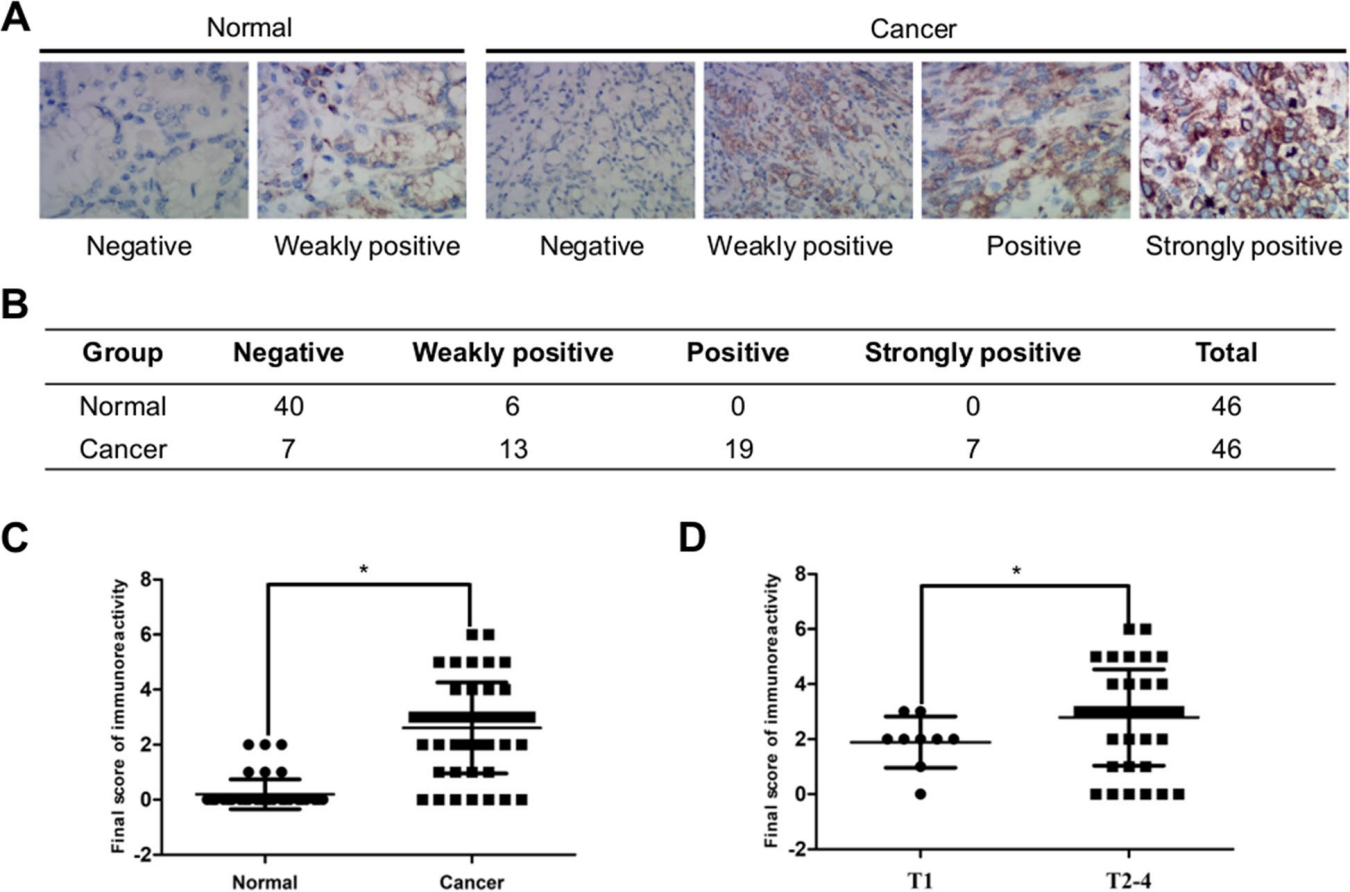
Expression of LUM in gastric cancer tissues. **A** LUM protein expression in representative samples of gastric cancer tissues and normal gastric tissues. **B**, **C** The statistic results of immunohistochemical staining of LUM. **D** The average staining scores of LUM expression in patients with early-stage (pathological stage T1) and advanced-stage (pathological stage T2-4) gastric cancer. *P < 0.05

IHC analysis was performed to investigate the location and intensity of LUM protein expression. The LUM protein expression was predominantly located in the cytoplasm of tumor cells, as observed through immunoreactivity. The IHC analysis for LUM showed weak to no expression of LUM protein in the neighboring noncancerous tissues of 46 patients. Specifically, weakly-positive LUM protein expression was observed in only 13% (6/46) of normal tissue samples (Fig. 6A, B). GC tissues showed levels of LUM expression that differed from those of non-cancerous adjacent tissues (Fig. 6A-C), with 84.8% (39/46) of tumor specimens displayed LUM protein expression but exhibited a wide range of variation, from weak to very strong expression. Fewer than 16% (7/46) of tumor specimens displayed negative LUM expression. Further analysis showed that expression of LUM was significantly correlated with T stage (Fig. 6D). Cumulatively, our results revealed that the overexpression of LUM might associate with tumor progression.

### Gene mutation analysis of LUM

**Fig. 7:**
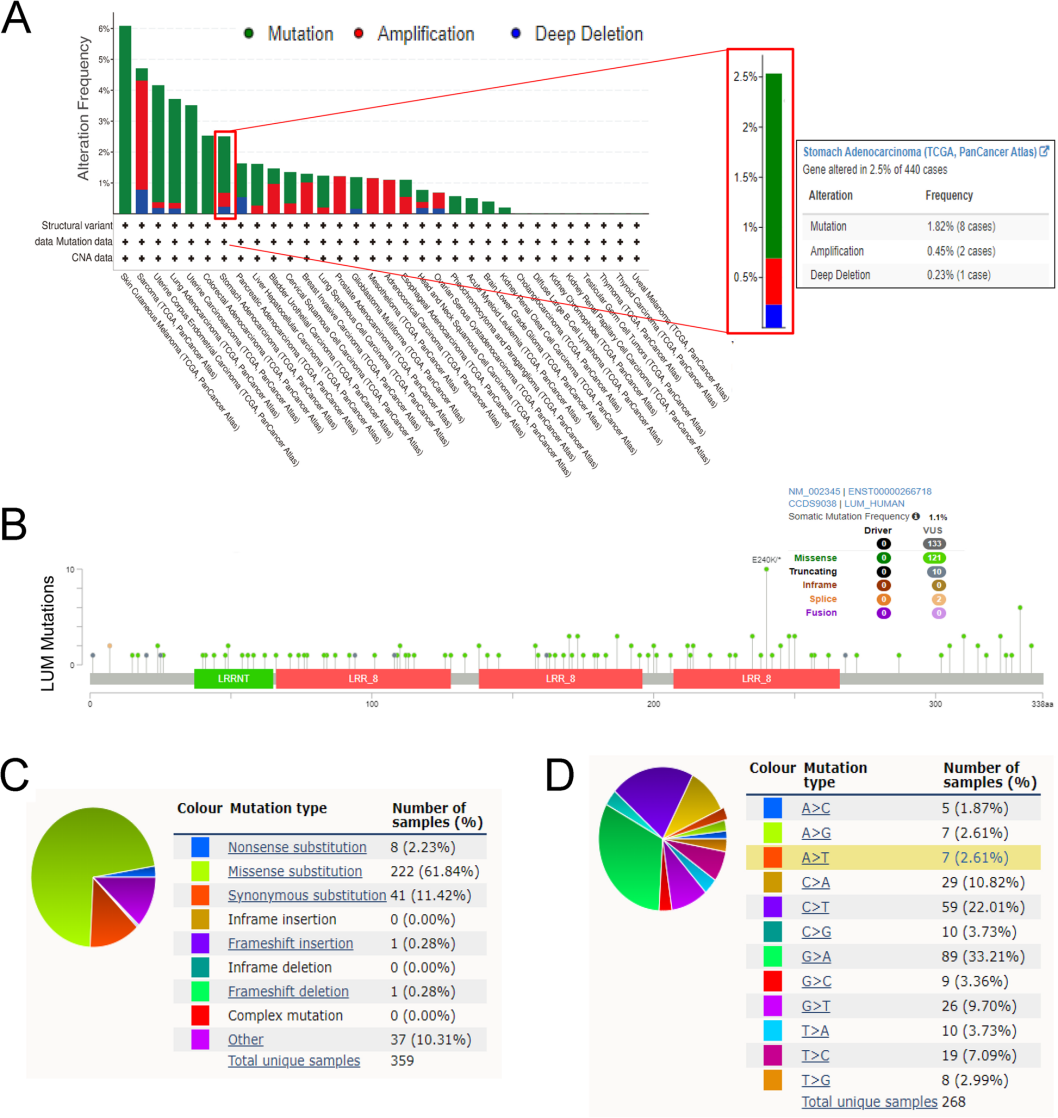
Mutation analysis of LUM in the cBioPortal database. **A** LUM alteration in pan-cancers. **B** Types of LUM mutation in pan-cancers. **C** Types and substitution of LUM mutation in pan-cancers

We queried the gene mutation sites of the LUM in the TCGA-STAD through the cBioPortal database and analyzed the results. According to the results (Fig. 7A), the main type of its genetic alterations was "mutation", which was observed in the majority of TCGA cancers, while “amplification” was the second most common. The “deep deletion” type in cancers was rare. We further explored the specific mutation type and site of LUM among cancers (Fig. 7B). Additionally, using the COSMIC online tool, we obtained an overview of the mutation types. The primary identified mutation type was missense substitution (61.84%), consistent with the findings from cBioPortal. The primary substitution mutation types were G > A (33.21%) and C > T (22.01%) (Fig. 7C, D).

### Diagnostic and prognostic significance of LUM expression

**Fig. 8:**
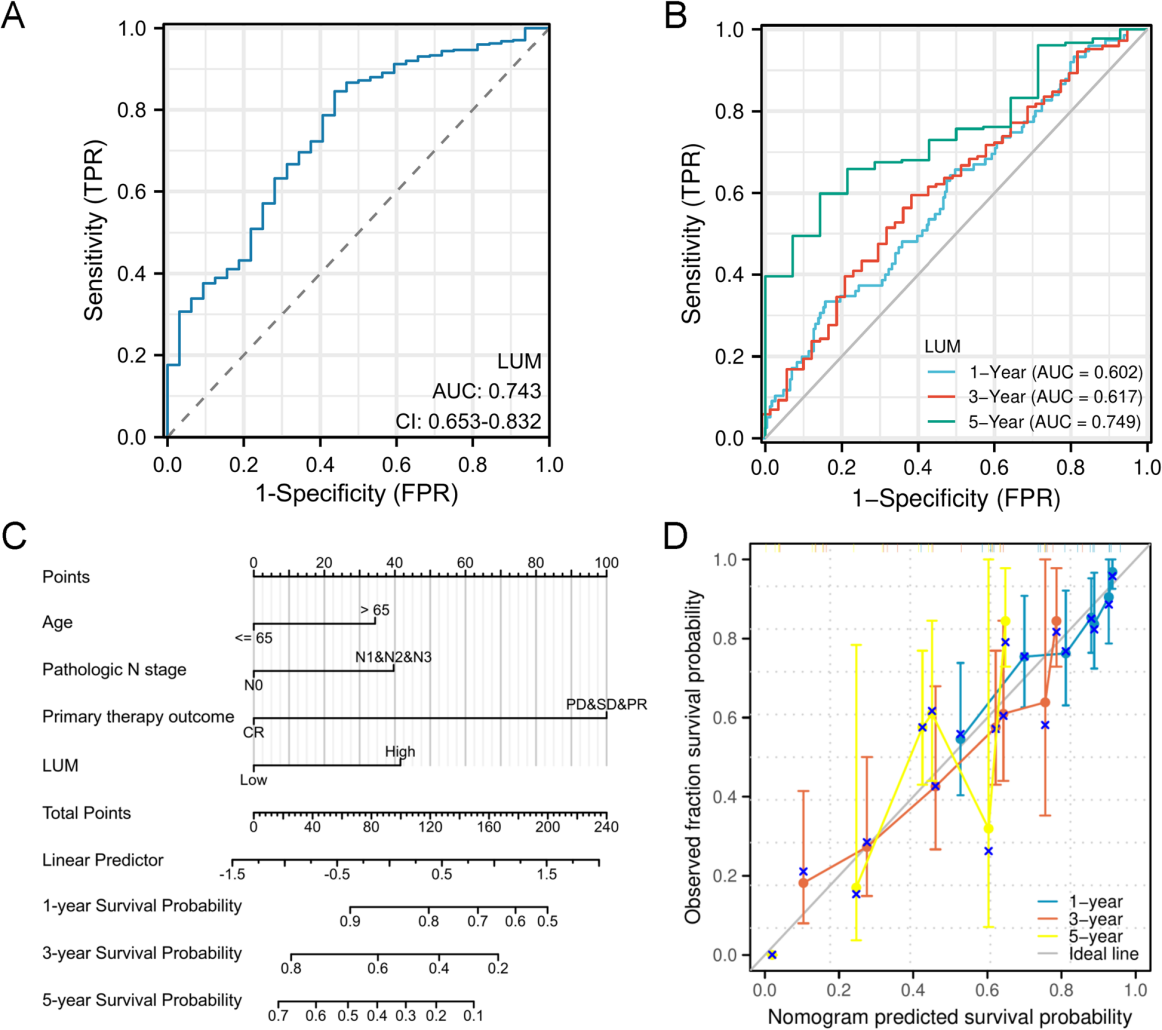
Relative operating characteristics (ROC) analysis and nomogram model of LUM. **A** Distinguishing tumor from normal tissue through ROC curve of diagnosis. **B** The prediction of 1-, 3-, and 5-year survival rates through time-dependent survival ROC curve analysis. **C** Nomogram plot to predict 1-, 3- and 5-year overall survival (OS). **D** Calibration curves of the nomogram to predict 1-, 3- and 5-year OS

The elevated expression level of LUM was considered to be an independent poor prognosis-associated factor linked to OS in STAD patients. Therefore it was assessed whether LUM expression could diagnose and predict prognosis in STAD patients. The area under the ROC curve (AUC) was 0.743 (95% CI: 0.653 − 0.832), indicating a moderate diagnostic value (Fig. 8A). This data indicated the function of LUM as a potential independent biomarker for differentiating STAD from non-tumor tissue. The survival rates of patients regarding 1-, 3-, and 5-year duration were predicted utilizing the time-dependent survival ROC curve of LUM. The AUC values of the ROC curve for OS prediction of the aforementioned durations were greater than 0.6, suggesting competent predictive performance (Fig. 8B). A nomogram model has been developed by integrating age, N stage, primary treatment outcome, and LUM level, which can be utilized to forecast the survival probability of patients in clinical practice for 1-, 3-, and 5-year (Fig. 8C). The concordance index (C-index) of the nomogram was 0.708 (95% CI, 0.682–0.733). The calibration curve demonstrates the reliable and accurate predictive power of the nomogram across the 1-, 3- and 5-year OS (Fig. 8D).

### LUM co-expression network

**Fig. 9:**
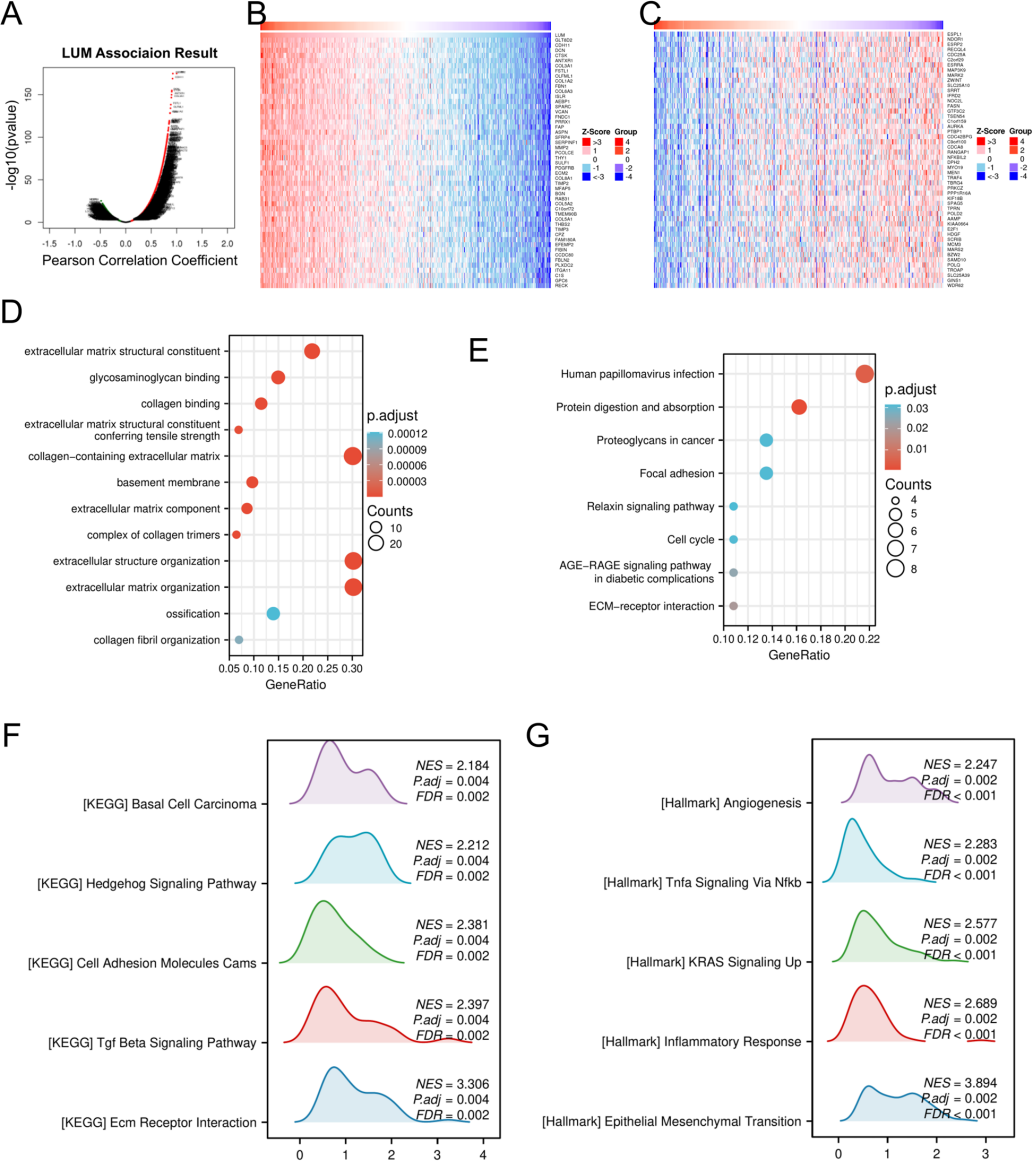
Enrichment analysis of LUM functional networks in stomach adenocarcinoma (STAD). **A** Pearson’s test identified LUM-related genes in the STAD cohort. **B** The significant 50 top genes positively linked to LUM are shown in the heat map. **C** The significant 50 genes that are most negatively linked to LUM are shown in the heat map. **D** Gene ontology analysis of LUM and its highly correlated genes in the STAD cohort. **E** Kyoto Encyclopedia of Genes and Genomes enrichment analysis of LUM and its highly correlated genes in STAD cohort. **F**-**G** Enrichment analysis of LUM expression-related gene set enrichment analysis (GSEA)

The database, LinkedOmics was employed to evaluate the LUM co-expression in STAD to understand better the biological significance of LUM in STAD under the parameters of p.adj 0.05. A total of 5,593 genes showed a positive correlation with LUM, while 3,894 genes negatively correlate with LUM (FDR < 0.01) (Fig. 9A). The 50 most positively and negatively linked genes to LUM were depicted utilizing a heat map (Fig. 9B, C). The GO function and KEGG enrichment analysis of LUM-related genes were carried out through the R software package. The 8 KEGG and GO functions numbering 81, 22, and 23 were identified regarding the biological processes (GO-BP), cellular components (GO-CC), and molecular functions (GO-MF), respectively. The top 12 GO terms (including 4 pieces of BP, CC, and MF) and the top 8 KEGG pathways, respectively were studied (Fig. 9D, E). As per GO term annotation, the DEGs associated with LUM are primarily involved in the organization of extracellular matrix (ECM) and extracellular structure, as well as, basement membrane, collagen-containing ECM, glycosaminoglycan binding, and ECM structural constituents (Fig. 9D). KEGG pathway analysis showed enrichment in human papillomavirus infections, proteoglycans in cancer, the digestion and absorption of proteins, the cell cycle, and other areas (Fig. 9E).

We performed GSEA enrichment analysis between the high-risk (> mean expression levels of LUM) and low-risk (< mean expression levels of LUM) groups in TCGA-STAD project. The GSEA enrichment analysis revealed that LUM was involved in the regulation of many cancer metabolics and cancer immune signaling pathways. Regarding the KEGG signaling pathways, the ecm receptor interaction, TGF-β signaling pathway, cell adhesion molecule cams, hedgehog signaling pathway, basal cell carcinoma, adipocytokine signaling pathway, etc. were differentially enriched in phenotypes with high LUM expression (Fig. 9F). Regarding the HALLMARK pathways, the epithelial mesenchymal transition, inflammatory response, kras signaling up, TNF-α signaling via NF-κB, angiogenesis, etc. were differentially enriched in phenotypes with high LUM expression (Fig. 9G). These results suggest that LUM plays a crucial role in tumorigenesis and progression.

### Immune signatures in correlation with LUM expression

**Fig. 10:**
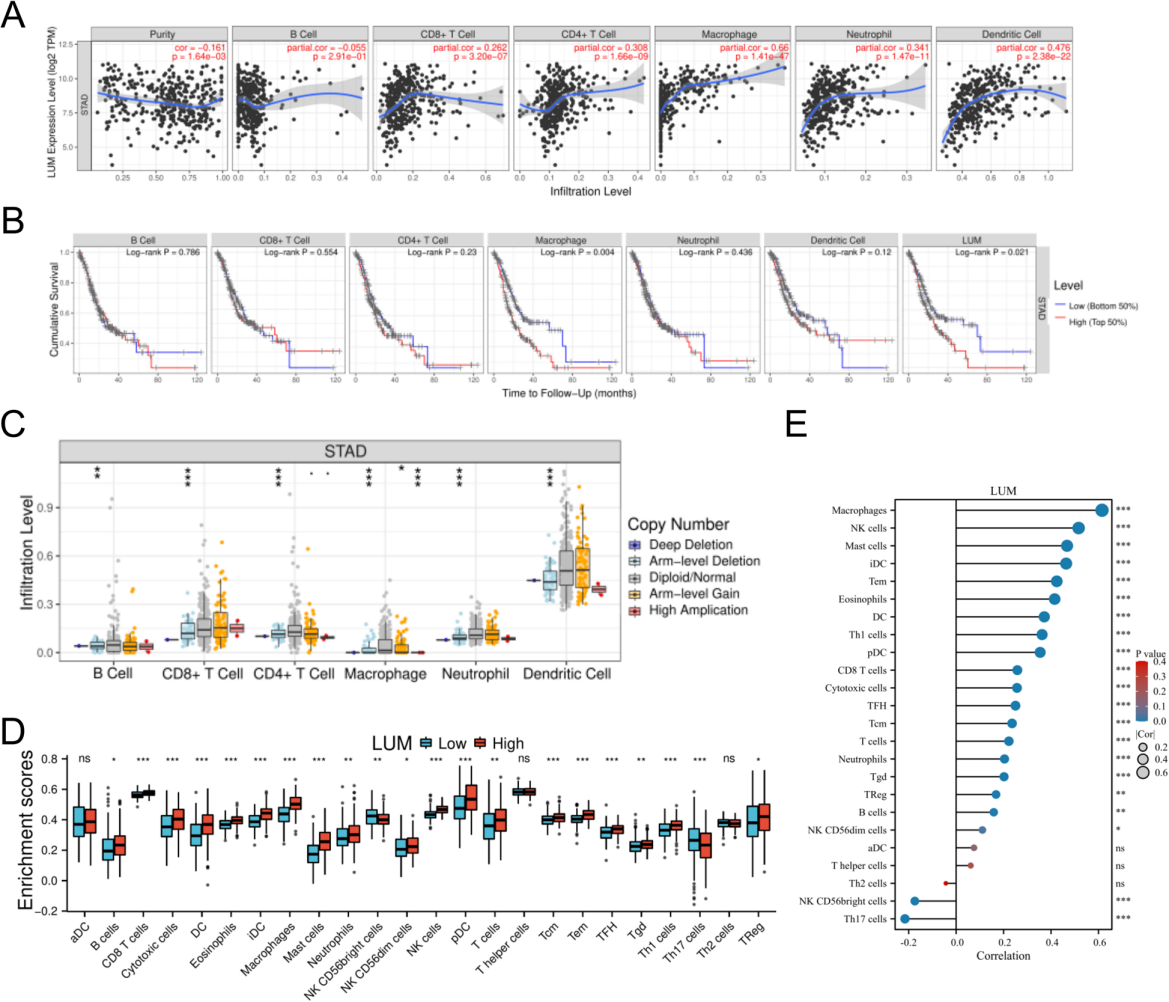
Link between LUM expression and the level of infiltration of immune cells in stomach adenocarcinoma (STAD). **A** Correlation between LUM expression and immune cell infiltration level. **B** Correlation of immune cell infiltration with the prognosis of patients with STAD. **C** The correlation between copy number alteration of LUM and immune cell infiltration in STAD. **D** The variation in the ratio of 24 immune cell subtypes in the low and high LUM expression groups in STAD tumor samples. **E** Correlation between LUM expression and 24 immune cells. * *P* < 0.05; ** *P* < 0.01; *** *P* < 0.001; and **** *P* < 0.0001. ns, not significant

The potential for increased expression of tumor-infiltrating lymphocytes as a prognosis-associated marker in individuals with GC has been demonstrated in some research reports [26–28]. As such, the TIMER was employed to analyze whether the LUM production in STAD is linked to the infiltration of immune-associated cells. The LUM expression was inversely linked to purity (*P* = 1.64E-13) and positively linked to CD8 + T cells (*P* = 3.20E-07), CD4 + T cells (*P* = 1.66E-09), macrophages (*P* = 1.41E-47), neutrophils (*P* = 1.47E-11) and dendritic cells (DCs) (*P* = 2.38E-22) (Fig. 10A). This data demonstrates that LUM performs a crucial function in STAD infiltration of immune cells. Cumulative survival concluded that macrophages of immune infiltrates of LUM in STAD were statistically significant (*P* < 0.05) indicating that macrophages substantially affect patients’ prognosis (Fig. 10B). Furthermore, different copy states of LUM were found to have a significant correlation with the infiltration status of immune cells such as CD8 + T cells, CD4 + T cells, B cells, neutrophils, macrophages, and DCs (Fig. 10C).

To examine the link between LUM and the degree of infiltration of immune cells in STAD, the GEPIA and TIMER databases were examined to gain a deeper insight into the interplay between LUM and various sets of immune markers, which were widely acknowledged to represent distinct immune cells, including B cells, T cells (general), CD8 + T cells, M1/M2 macrophages, tumor-associated macrophages (TAMs), neutrophils, natural killer (NK) cells, monocytes, and DCs in STAD (Table 5). In addition, various functional T cells were analyzed, including T-helper 1 (Th1) cells, Th2 cells, Th17 cells, follicular helper T cells (Tfh), exhausted T cells, and Treg. The findings revealed that the level of the majority of the immune sets marking various T cells, B cells, M1/M2 macrophages, TAMs, neutrophils, NK cells, DCs, and monocytes were linked to the expression of LUM in STAD. Therefore, the analysis indicates the link of the infiltration level of these cells to the expression of LUM in STAD in a variety of ways.

**Table 5:** Correlation analysis between LUM and relate genes and markers of immune cells in TIMER and GEPIA

Description	Gene markers	TIMER				GEPIA			
		None		Purity		Tumour		Normal	
		Cor	P	Cor	P	R	P	R	P
CD8 + T cell	CD8A	0.274	***	0.251	***	0.23	***	-0.065	0.710
	CD8B	0.142	**	0.129	*	0.12	*	-0.097	0.570
T cell (general)	CD3D	0.244	***	0.211	***	0.19	***	-0.28	0.100
	CD3E	0.242	***	0.211	***	0.22	***	-0.25	0.130
	CD2	0.32	***	0.3	***	0.3	***	-0.2	0.250
B cell	CD19	0.19	***	0.176	***	0.14	**	-0.16	0.370
	CD79A	0.233	***	0.201	***	0.19	***	-0.38	*
Monocyte	CD86	0.496	***	0.473	***	0.47	***	-0.023	0.890
	CD115(CSF1R)	0.555	0	0.53	***	0.51	***	0.18	0.300
TAM	CCL2	0.541	0	0.519	***	0.52	***	0.53	**
	CD68	0.305	***	0.269	***	0.28	***	-0.5	**
	IL10	0.494	***	0.476	***	0.48	***	0.25	0.150
M1 Macrophage	INOS(NOS2)	0.029	0.562	0.02	0.697	0.042	0.400	0.2	0.250
	IRF5	0.257	***	0.247	***	0.26	***	-0.54	***
	COX2(PTGS2)	0.297	***	0.307	***	0.32	***	0.68	***
M2 Macrophage	CD163	0.507	0	0.477	***	0.49	***	0.67	***
	VSIG4	0.558	0	0.536	***	0.53	***	0.57	***
	MS4A4A	0.579	0	0.559	***	0.55	***	0.72	***
Neutrophils	CD66b(CEACAM8)	0.016	0.746	0.038	0.465	0.045	0.360	0.27	0.100
	CD11b(ITGAM)	0.496	0	0.48	***	0.47	***	0.49	**
	CCR7	0.27	***	0.238	***	0.24	***	-0.4	*
Natural killer cell	KIR2DL1	0.179	***	0.184	***	0.14	**	0.18	0.300
	KIR2DL3	0.095	0.053	0.083	0.107	0.17	***	0.08	0.640
	KIR2DL4	0.019	0.694	-0.012	0.808	-0.0078	0.880	-0.35	*
	KIR3DL1	0.118	*	0.113	*	0.099	*	0.35	*
	KIR3DL2	0.155	**	0.146	**	0.14	**	-0.07	0.690
	KIR3DL3	-0.088	0.072	-0.081	0.171	-0.1	*	-0.23	0.170
	KIR2DS4	0.07	0.152	0.06	0.243	0.034	0.500	0.17	0.310
Dendritic cell	HLA-DPB1	0.326	***	0.29	***	0.28	***	-0.064	0.710
	HLA-DQB1	0.174	***	0.133	**	0.11	*	-0.22	0.200
	HLA-DRA	0.276	***	0.243	***	0.26	***	-0.093	0.590
	HLA-DPA1	0.284	***	0.249	***	0.24	***	-0.099	0.560
	BCDA-1(CD1C)	0.372	***	0.351	***	0.35	***	-0.095	0.580
	BDCA-4(NRP1)	0.632	0	0.618	***	0.6	***	0.78	***
	CD11c(ITGAX)	0.484	0	0.455	***	0.45	***	0.15	0.390
Th1	T-bet(TBX21)	0.234	***	0.221	***	0.20	***	-0.052	0.760
	STAT4	0.342	***	0.334	***	0.33	***	0.065	0.710
	STAT1	0.102	*	0.093	0.069	0.11	*	0.3	0.078
	IFN-γ(IFNG)	0.069	0.158	0.062	0.229	0.072	0.140	-0.03	0.860
	TNF-α(TNF)	0.137	**	0.109	*	0.15	**	-0.32	0.058
Th2	GATA3	0.323	***	0.314	***	0.27	***	-0.31	0.620
	STAT6	0.109	*	0.102	*	0.11	*	0.31	0.077
	STAT5A	0.386	***	0.384	***	0.35	***	0.72	***
	IL13	0.137	**	0.165	**	0.18	***	0.33	0.053
Tfh	BCL6	0.377	***	0.353	***	0.34	***	0.43	**
	IL21	0.126	*	0.108	*	0.13	**	-0.15	0.400
Th17	STAT3	0.346	***	0.335	***	0.32	***	0.36	*
	IL17A	-0.134	**	-0.15	**	-0.1	*	-0.31	0.064
Treg	FOXP3	0.278	***	0.253	***	0.27	***	-0.44	**
	CCR8	0.407	***	0.4	***	0.39	***	-0.32	0.060
	STAT5B	0.445	0	0.442	***	0.46	***	0.71	***
	TGFβ(TGFB1)	0.542	***	0.528	***	0.5	***	0.049	0.780
T cell exhaustion	PD-1(PDCD1)	0.17	***	0.151	**	0.14	**	-0.16	0.360
	CTLA4	0.186	***	0.164	**	0.19	***	-0.31	0.063
	LAG3	0.147	**	0.122	*	0.11	*	0.11	0.530
	TIM-3(HAVCR2)	0.503	0	0.482	***	0.47	***	0.21	0.220
	GZMB	0.15	**	0.11	*	0.12	*	-0.15	0.390

TIMER The tumor immune estimation resource, *GEPIA* Gene expression profiling interactive analysis, *CD* Cluster of differentiation, *TAM* Tumor-associated macrophage, *Th* T helper cell, *Tfh* Follicular helper T cell, *Treg* Regulatory T cell, *Cor* R value of Spearman's correlation, None Correlation without adjustment, *Purity* correlation adjusted by purity. * *P* < 0.05; ** *P* < 0.01; *** *P* < 0.001

Furthermore, 24 of the infiltrating immune cells were compared in different LUM expression groups to determine whether these cells exhibited variation in expression due to the LUM expression. The tumor immune microenvironment (TIME) was examined for any difference between low and high LUM expression levels in STAD. Except for aDC, T helper cells, and Th2 cells, this result suggests that most immune infiltrating cells depicted variation in their expression between the two LUM expression groups (Fig. 10D). Therefore, it can be concluded that the TIME differs in STAD between low and high LUM expression levels. Subsequently, we performed a correlation analysis between the scores of 24 immune cells and the expression of LUM. The lollipop plot showed that, in addition to Th2 cells, T helper cells, and aDC, the expression of LUM was significantly positively correlated with the infiltration of multiple immune cells (Fig. 10E).

### LUM-related ceRNA network construction in STAD

**Fig. 11:**
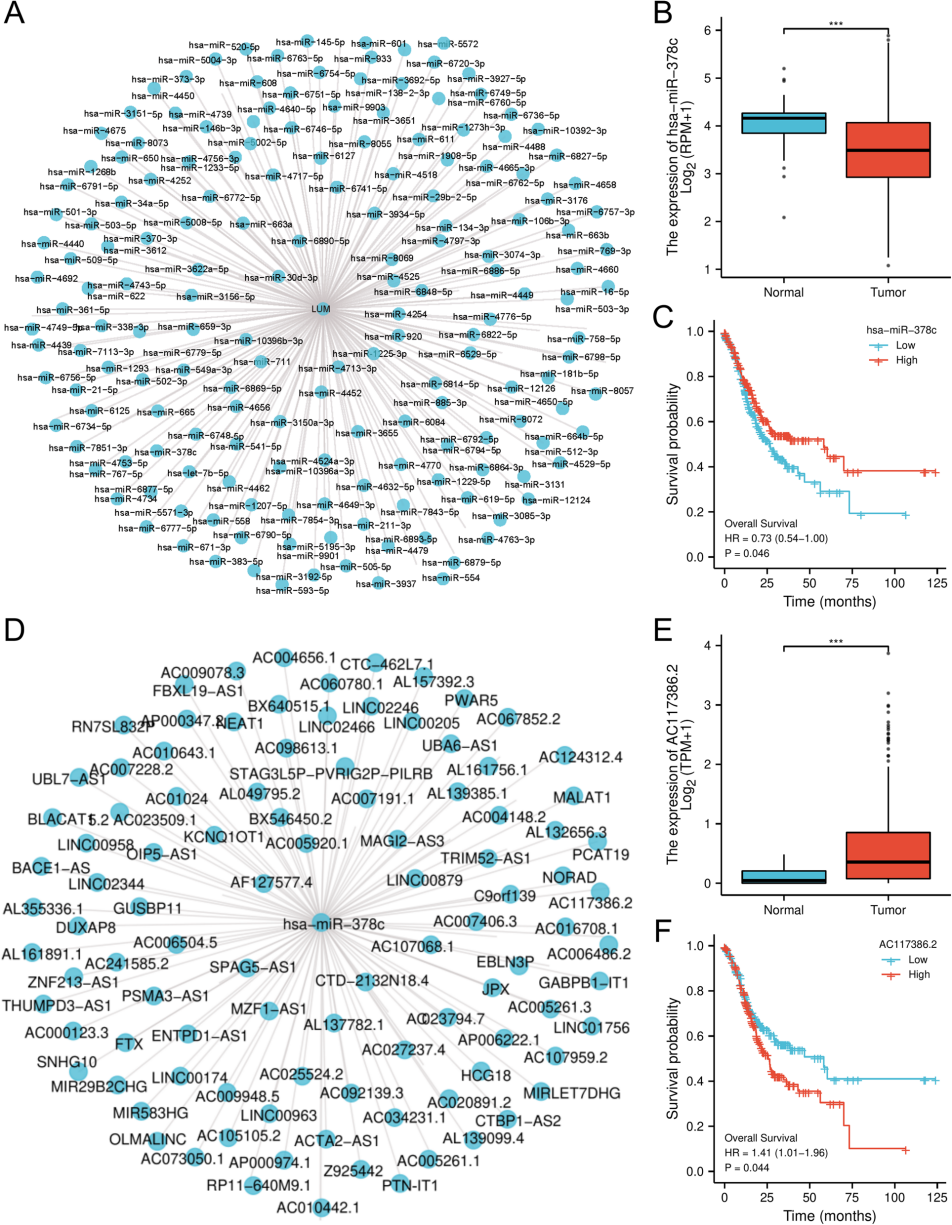
Development of lncRNA-miRNA-mRNA regulatory axis. **A** The miRNA-LUM network is established using Cytoscape. **B** The expression level of has-miR-378c in the STAD cohort. **C** The prognostic value of has-miR-378c in the STAD cohort. **D** The lncRNA-has-miR-378c network is established using Cytoscape. **E** The expression level of AC117386.2 in the STAD cohort. **F** The prognostic value of AC117386.2 in the STAD cohort

The link of the mRNA-miRNA-lncRNA-associated ceRNA network to several human cancers was established to develop a ceRNA network involving LUM in STAD. Using the miRWalk database, upstream miRNAs that may interact with LUM were predicted, yielding a total of 170 miRNAs (Supplementary file 2). A miRNA-LUM regulatory network was developed utilizing the Cytoscape software to improve the presentation of the results (Fig. 11A). It was hypothesized that there should be a negative correlation between miRNA and LUM based on the action mechanism of miRNA-mediated gene regulation. The findings of the study showed that has-miR-378c was downregulated in STAD (Fig. 11B), and STAD patients with high hsa-miR-378c levels had significantly longer OS (Fig. 11C). Based on this finding, its upstream lncRNA targets were investigated to build the miRNA–lncRNA axis. The hsa-miR-378c was submitted to StarBase and the results suggested that the identified 100 long noncoding RNAs (lncRNAs) had the potential lncRNA targets of hsa-miR-378c. Similarly, to improve the presentation of the results, a lncRNA-hsa-miR-378c regulatory network was developed using the Cytoscape software (Fig. 11D). A variety of lncRNAs were examined in detail (Supplementary file 3). As per the ceRNA mechanism, these lncRNAs should act as oncogenes in STAD. Only AC117386.2 was considerably upregulated in STAD specimens and was linked to poor survival in STAD patients (Fig. 11E, F). Thus, the AC117386.2/hsa-miR-378c/LUM regulatory axis may be the most likely mechanism in mediating STAD tumor stage progression.

## Discussion

GC is notorious for its ability to metastasize to regional lymph nodes, the lungs, the liver, and the peritoneal cavity, all of which always contribute to a poor patient’s prognosis. Although there have been considerable developments in the molecular basis, diagnosis, and treatment of GC, most of the patients when being diagnosed have already reached the advanced stage. Exploring the underlying molecular mechanisms of GC development is tremendously valuable and important for identifying new potential therapeutic targets and thus further improving the prognosis of GC.

This study combined GEO and TCGA data and showed that the levels of expression of ITGB1, LUM, and COL5A2 were considerably upregulated in both early and advanced GC tissues and correlated with a shorter survival time in GC patients. Furthermore, pathologic stage analysis revealed that when the LUM and COL5A2 genes were overexpressed, the pathologic stage of GC patients was relatively high. Numerous studies have depicted these core genes as having carcinogenic properties or acting as potential cancer surveillance biomarkers. Xu et al*.* found that ITGB1 acts as a cancer gene in esophageal squamous cell carcinoma, contributing to apoptosis reduction and promoting cell migration and invasion by activating the FAK-Rac1 pathway [29]. Through bioinformatics analysis of RNA sequencing data, Iwatate et al*.* discovered that ITGB1 is linked to pancreatic cancer metastasis, progression, and prognosis [30]. Yang et al*.* found that suppressing LUM increased the cell-doubling time and resulted in the suppression of cell growth in lung cancer cell lines [31]. Zang et al. identified over-expression of LUM as a potential novel target for colorectal adenocarcinoma [32]. Ding et al. found that increased COL5A2 expression is strongly linked to poor prognosis and renal tumor metastasis in GC patients [33]. Zeng et al*.* found that COL5A2 may predict poor clinical outcomes in bladder cancer patients, and its increased production is significantly linked to a lower survival rate [34]. All these reports, together with this research, strongly suggest that ITGB1, LUM, and COL5A2 are linked to GC progression.

The identification of independent prognostic factors is critical for many patients with GC and may guide clinical treatment. Therefore, the search for an independent prognostic factor has become a top priority in oncology research. The accurate selection of prognostic factors depended upon the correct identification of the independent prognosis-associated markers utilizing multivariate analyses by employing the Cox proportional hazards model. The findings demonstrated that increased expression of LUM serves as an independent and unfavorable prognostic factor affecting the survival of patients with GC and that it has the potential to become a target for therapeutic interventions as well as act as a prognosis-associated biomarker. This is in line with the findings of prior research [35, 36]. Furthermore, we verified the results by collecting clinical samples for IHC analysis, which made our research results more reliable. Based on these findings, the role of LUM expression levels was investigated in the diagnosis and prognosis of GC patients in this research. The expression of LUM was demonstrated to be capable of distinguishing tumors from normal tissue and predicting 1-, 3-, and 5-year OS rates in patients, highlighting its involvement as a clinical diagnostic and prognostic biomarker for GC.

LUM is a keratan sulfate proteoglycan that is part of the small leucine-rich proteoglycan family and is an ECM component [37, 38]. LUM is ubiquitously distributed in almost all tissues of the human body as a vital component of the ECM in the form of proteoglycan [39, 40]. Extensive research has revealed that ECM has a crucial function in tumor proliferation, invasion, and migration, as well as its potential as an anticancer therapeutic target [41–43]. Simultaneously, LUM regulates the proliferation, migration, and tube formation of primordial epithelial cells, contributing to tumor angiogenesis [44]. Inflammation responses in the tumor microenvironment are an integral component of the tumor-associated immune response and have a vital role in the onset and progression of cancer [45, 46]. Previous studies have substantiated that LUM is involved in tumor cell biology via modulating tumor inflammatory signal transduction [47, 48].

The potential mechanism by which LUM influences cancer development and progression is complex and unknown. Co-expression analysis is widely used to infer putative gene function and aid in determining the roles of the genes within phenotypic differences [49]. Prior to this manuscript, there were no reports on the functional enrichment analysis of LUM co-expression in GC. A co-expression analysis and functional enrichment analyses were executed to further show the potential biological function of LUM in the GC microenvironment. Using the top 100 co-expressed LUM genes, GO analysis revealed that BP, CC, and MF are all involved in ECM development-related pathways and signal transduction. This suggests that LUM is important in the homeostasis of ECM components. A previous study found that ECM and ECM-related events had a strong influence on GC progression [50]. Deregulation of the ECM has been shown in studies to affect cancer progression and directly promote tumor cell metastasis [51]. KEGG analysis and GSEA enrichment analysis depicted that the genes co-expressed with LUM were enriched in several cancer-related pathways. Overall, this suggests that LUM could be involved in developing GC.

The tumor microenvironment is critical in the development and progression of cancer. Understanding the tumor microenvironment may thus aid in deciphering the regulatory mechanisms underlying tumor development. The tumor microenvironment is a dynamic and complex milieu of non-tumorous cells around tumor cells. In a previous study, changes in proteoglycan expression in tumor cells and the tumor microenvironment were linked to oncogenesis [52]. The tumor immune microenvironment is one of the major components of tumor microenvironment, consisting of various immune cells infiltrating the tumor [53]. Therefore, the link between LUM expression and tumor-infiltrating immune cells in the tumor microenvironment was examined. It was found for the first time that LUM expression was inversely linked with the levels of purity and positively linked to the levels of CD8 + T cells, CD4 + T cells, macrophages, neutrophils, and DCs in GC. Additionally, macrophage infiltration was found to have a significant association with GC prognosis in the current study. Macrophages constitute an important class of immune cells in the tumor immune microenvironment and their frequency is often associated with unfavorable patient survival [54]. Macrophages are involved in malignant processes such as cell invasion, angiogenesis and metastasis [55]. Recently, it has been confirmed that LUM expression level was implicated in macrophage-conditioned media (maCM)-induced cell invasion [47]. The possible biological mechanism may be as follows: tumor cells can secrete colony-stimulating factor and attract macrophages, which in turn secrete epidermal growth factor to guide tumor cells toward blood vessels [56, 57]. Furthermore, compared with normal tissue, different copy states of LUM have some effect on immune immersion. Based on these results and findings, it can be concluded that LUM affects the infiltration of the immune cells in the tumor microenvironment and contributes to poor prognosis in GC patients. Moreover, the discovery of a linkage between LUM expression and the expression of certain immunological marker genes suggests that LUM plays a role in controlling immune cell infiltration and regulating tumor immunity in STAD, although establishing a cause-effect relationship was not possible in the current study. Meanwhile, it was noted that most of the immune cells or their markers performed poorly in the prediction of correlations using the TIMER and GEPIA databases. A similar phenomenon has been observed in a recent study [58]. This weak link does not imply that the detected target molecule can be ignored. These findings also show that the TIME differs between low and high LUM expression levels in STAD. According to research findings, LUM overexpression in STAD patients influenced antitumor immune responses. While these findings show a link between LUM and immune response, more research is needed to elucidate possible links between the two.

The current focus on the pathogenesis of human diseases has led to the emergence of noncoding RNAs (ncRNAs) as hotspots of human genome research [59]. Extensive research has linked ncRNA dysregulation to numerous disorders, including cancer [60, 61]. In 2011, Salmena and colleagues first proposed a new regulatory mechanism between ncRNA and mRNA, namely the ceRNA hypothesis. Genes can be silenced through the binding of miRNAs to mRNAs, whereas lncRNAs can upregulate the target gene’s expression by competitively binding to miRNAs [62, 63]. Furthermore, the use of ncRNA as markers for early diagnosis, long-term survival prediction, and as a compelling therapeutic target has been confirmed by previous studies [64–66]. This research showed that the expression level of hsa-miR-378c was lowered when compared to adjacent normal tissue control in STAD. A survival analysis revealed that high hsa-miR-378c expression depicted a significant association with a favorable STAD prognosis. Consequently, the hsa-miR-378c may be considered to be a negative regulator of GC via its target LUM. It has been demonstrated that hsa-miR-378c could be a biomarker for the early diagnosis and prognosis of human cancers. For example, has-miR-378c can be used as a colorectal cancer biomarker in individuals with early-stage II colon cancer [67]. Ma et al*.* suggested that hsa-miR-378c has a significant diagnostic and prognostic value in patients with cervical squamous cell carcinoma [68]. These findings go some way to support the accuracy of the bioinformatics analysis in this experiment. Using the StarBase database 100 upstream potential lncRNAs interacting with hsa-miR-378c were identified. AC117386.2 was identified as the highly probable upstream lncRNA of the LUM/hsa-miR-378c axis in STAD using a combination of survival analysis and expression analysis for these miRNAs. AC117386.2’s Ensembl ID is ENSG00000243944. AC117386.2 is a novel lncRNA transcript and has been reported sparingly in the literature, and thus, it warrants further investigation. The AC117386.2/hsa-miR-378c/LUM axis has been identified as a potential regulatory pathway in STAD.

This research is the first attempt to comprehensively analyze the link between LUM expression, the infiltration of tumor-associated immune cells, and the ceRNA network in STAD. Undoubtedly, this research has some limitations. First, although differential expression of LUM was detected between early gastric cancer, advanced gastric cancer and tumor-adjacent tissues, the prognostic implication of this finding has not been demonstrated. Second, while the link between LUM and immune infiltration in STAD patients was explored, more research is needed to validate these findings. Moreover, the identification of the AC117386.2/hsa-miR-378c/LUM axis as having the potential to act as a regulatory pathway in STAD was possible through this work. However, in vivo and in vitro studies should confirm the AC117386.2/hsa-miR-378c/LUM regulatory axis. Nevertheless, it can be concluded that LUM is a promising therapeutic target for treating GC.

## Conclusions

This study is the first to identify LUM as a possible key oncogene in gastric cancer progression and the first to comprehensively analyze the relationship between LUM expression and tumor immune infiltration and ceRNA networks in gastric cancer. Besides, it has been found that the components of the AC117386.2/hsa-miR-378c/LUM network may be used as promising therapeutic targets and prognostic biomarkers in the future.

### Supplementary Information

**Additional file 1.** 

**Additional file 2.** 

**Additional file 3.**

## Data Availability

The results shown here are in whole or part based upon data generated by GEO (https://www.ncbi.nlm.nih.gov/geo/query/acc.cgi?acc=GSE3438) and TCGA database (https://portal.gdc.cancer.gov/projects/TCGA-STAD). The data used in the current study are available from the corresponding author upon reasonable request.

## Abbreviations

ABC: Avidin–biotin-enzyme complex
ADAM12: A disintegrin and metalloprotease 12
AGC: Advanced-stage gastric cancer
AJCC: American joint committee on cancer
ANXA2: Annexin A2
AUC: Area under the curve
BP: Biological processes
CC: Cellular components
CD44: Cluster of differentiation 44
ceRNA: Competing endogenous RNA
CI: Confidence interval
C-index: Concordance index
COL3A1: Collagen type III alpha 1 chain
COL5A2: Collagen type V alpha 2 chain
CTSB: Cathepsin B
DCs: Dendritic cells
DEGs: Differentially expressed genes
ECM: Extracellular matrix
EGC: Early-stage gastric cancer
FC: Fold change
FDR: False discovery rate
GC: Gastric cancer
GEO: Gene expression omnibus
GEPIA: Gene expression profiling interactive analysis
GO: Gene ontology
COSMIC: Catalogue of somatic mutations
GSEA: Gene set enrichment analysis
GSVA: Gene set variation analysis
GTEx: Genotype-tissue expression
HR: Hazard ratio
HSP90AA1: Heat shock protein 90-alpha
IHC: Immunohistochemistry
ITGB1: Integrin subunit β1
KEGG: kyoto encyclopedia of genes and genomes
K-M: Kaplan–Meier
lncRNA: Long non-coding RNA
LUM: Lumican
MCC: Maximal Clique Centrality
MF: Molecular functions
miRNA: Micro RNA
mRNA: Messenger RNA
ncRNAs: Noncoding RNAs
NK: Natural killer
OS: Overall survival
PCA: Principal component analysis
PPI: Protein–protein interaction
ROC: Relative operating characteristics
SCNA: Somatic copy number alterations
SERPINH1: Serpin peptidase inhibitor clade H, member 1
ssGSEA: Single sample genome enrichment analysis
STAD: Stomach adenocarcinoma
STRING: Search tool for the retrieval of interacting genes
TAMs: Tumor-associated macrophages
TCGA: The cancer genome atlas
Tfh: Follicular helper T cells
Th1: T-helper 1
Th17: T-helper 17
Th2: T-helper 2
TIME: The tumor immune microenvironment
TIMER: The tumor immune estimation resource
TIMP1: Tissue inhibitor of metalloproteinase-1
TIMP2: Tissue inhibitor of metalloproteinase-2
TNM: Tumor-node-metastasis
TPM: Transcripts per million
